# Anxiety, Depression and Risk of Post-Traumatic Stress Disorder in Health Workers: The Relationship with Burnout during COVID-19 Pandemic in Italy

**DOI:** 10.3390/ijerph18189929

**Published:** 2021-09-21

**Authors:** Lucio Ghio, Sara Patti, Giulia Piccinini, Cinzia Modafferi, Eleonora Lusetti, Massimo Mazzella, Massimo Del Sette

**Affiliations:** 1 Psychiatry Branch, Galliera Hospital, Mura delle Cappuccine, 14, 16128 Genoa, Italy; lucio.ghio@asl3.liguria.it (L.G.); giulia.piccinini@asl3.liguria.it (G.P.); eleonora.lusetti@asl3.liguria.it (E.L.); 2 V.I.E. Development, Innovation, Empowerment srl Spinoff, University of Genoa, Via Eugenia Ravasco 12, 16128 Genoa, Italy; modafferi@vie-srl.it; 3 Maternal and Infant Department, Galliera Hospital, Mura delle Cappuccine, 14, 16128 Genoa, Italy; massimo.mazzella@galliera.it; 4 Neurology Unit, Galliera Hospital, Mura delle Cappuccine, 14, 16128 Genoa, Italy; massimo.del.sette@galliera.it; 5 San Martino Polyclinic Hospital IRCCS, Largo R. Benzi, 10, 16132 Genoa, Italy

**Keywords:** COVID-19, healthcare, healthcare workers, burnout, post-traumatic stress disorder, psychological trauma, depression, risk factors, anxiety, pandemic

## Abstract

During the COVID-19 pandemic, healthcare workers (HW) have faced an extremely difficult work environment, with an increased workload and traumatic events. Our study aimed to investigate the impact of COVID-19 pandemic on HW’s mental wellbeing. We analyzed the correlations between levels of burnout and other mental health disorders and we searched for the presence of specific risk factors of post-traumatic symptomatology related to the pandemic. A structured an on-line questionnaire and validated instruments were completed by a sample of HW from some hospitals in Genoa, Italy. Anxious, depressive, post-traumatic and other psychological symptoms were assessed and risk factors, related to the pandemic, were considered. Then, we investigated the correlation between levels of burnout and the risk of developing psychopathology. A total of 731 HW were screened, and we found increased levels of anxiety (61%), depression (62%), PTSD (34%) and high levels of burnout; especially emotional exhaustion (37%). A statistically significant association between burnout and insomnia, depression, anxiety, and post-traumatic symptoms was demonstrated. This study indicates that during the COVID-19 pandemic, HW showed high levels of psychological distress and that burnout is an important predictor of sufferance. These findings support the idea to provide psychological and psychiatric support for HW.

## 1. Introduction

During the first Italian peak, in spring 2020, many major cities in our country faced overloaded intensive care units and emergency departments due to a high hospitalization for Sars-CoV2 disease. In Genoa, a city of almost 600,000 inhabitants, with 27.2% of the population over sixty-five years old, the daily hospitalization reached the peak of 719, 13% of those were admitted to intensive care units [1]. By 30 April 2020, Genoa faced 663 deaths because of COVID-19 disease: the death rate of the city, compared with the mean rates of the previous five years, increased by 56.1% in March 2020 and by 71.6% in April 2020 [2]. From mid-March 2020, most of the main hospitals in Genoa were converted by the regional government into “COVID-19 hospitals”, where many wards were turned into areas dedicated to the treatment of COVID-19 patients.

The COVID-19 pandemic has revealed that healthcare workers (HW) are at risk of developing psychological and psychiatric disorders due to the extreme working conditions [3], with a high rate of anxiety, depression, and insomnia among them [4,5,6,7]. These results were already suggested by literature on previous large-scale disasters, which pointed out that a high percentage of HW could experience post-traumatic stress disorder (PTSD), depression, anxiety, and substance disorders during these sanitary emergencies [8].

Literature on the COVID-19 pandemic has pointed out that there are many specific risk factors that can predict worse outcomes regarding HW’s mental health status, such as the fear of being infected and/or to infect loved ones, higher exposure to death, role and less job seniority, younger age, female gender, absence of social support, and being frontline workers [2,9,10,11,12,13,14]. In addition, HW are at risk of experiencing high baseline burnout rates that were exacerbated by the increased levels of stress which occurred during thi pandemic [3,15].

Indeed, burnout and PTSD are both frequent syndromes in professionals working in emergency settings due to external stressors, such as work-related traumatic experiences [16], and they also share similar drivers, consequences, and comorbidities [17].

A previous study investigated the correlation between PTSD and burnout in an Italian sample of emergency HW before the COVID-19 pandemic and found a positive correlation between the two constructs [16].

During this pandemic, burnout was identified among HW and persistent burnout was found to be able to contribute to higher levels of anxiety, acute stress, and depersonalization symptoms [18,19,20].

Therefore, the aim of our study is to investigate the prevalence of PTSD, insomnia, anxiety, and depression on a population of HW highly involved in the pandemic emergency, and to evaluate if these disorders are correlated to specific COVID-19 risk factors, to better understand their specific trajectories, development, and duration in this population. Moreover, unlike most research, we decided to analyze levels of burnout in order to obtain a more comprehensive picture of HW’s psychological wellbeing, trying to underline the correlation between high levels of burnout and other mental health disorders.

## 2. Material and Methods

### 2.1. Study Design and Sample

This study collects the baseline data of a larger longitudinal project on HW’s mental health, which included a prompt response to their sufferance offering both psychiatric and psychological support within the hospitals.

The baseline survey was fillable online from 30 July 2020 to 30 September 2020 (about three months after the first COVID-19 peak in our country) and the second assessment was repeated after six months.

The survey was approved by the regional ethics committee (CER, Commissione etica Regionale, n.299/2020).

We analyzed baseline data collected with the survey administered to a sample of HW from Galliera, Scassi General Hospitals, and other minor hospitals of the city, that was filled online on the Lime Survey platform. All personnel working during the peak of the pandemic were asked to participate in the study, but the enrollment was voluntary, anonymous and participants could withdraw at any moment.

### 2.2. Study Instrument

The survey, designed as a structured questionnaire, was delivered on the Lime Survey platform and investigated sociodemographic characteristics, occupational variables, personal exposures to COVID-19 and validated instruments were included to assess psychological distress. The order of sociodemographic questions and of the questionnaires were randomized to minimize the bias related to the order of presentation. It required about 10–15 min to be completed.

#### 2.2.1. Depressive Symptoms

Symptoms of depression were evaluated with the Patient Health Questionnaire-9 (PHQ-9), which has shown a high internal consistency (Cronbach’s α = 0.83) [21,22]. It is a self-administered questionnaire with 9 items that requires the participant to evaluate, on a 4-points Likert scale, the intensity of the symptoms in the last two weeks. There is a tenth item that evaluates the functional impairment caused by depression and explores to what extent the depression made it difficult to work and to maintain relationships.

The first 9 items contribute to the total score, which helps to stratify different severity levels of the depression (range 0–27; cut-off points of 5, 10, 15, 20 might be interpreted as representing mild, moderate, moderately severe, and severe depression).

#### 2.2.2. Generalized Anxiety Disorder Symptoms

Symptoms of anxiety were assessed using the Generalized Anxiety Disorder-7 (GAD-7), which has demonstrated good psychometric properties, including sensitivity and specificity for diagnostic GAD and a good reliability (Cronbach’s α = 0.89) [23]. The instrument is self-administered and made of seven items that assess symptoms of GAD experienced over the past two weeks on a 4-points Likert scale (range 0–21; cut-off of 5, 10 and 15 might be interpreted as representing mild, moderate, and severe levels of anxiety).

#### 2.2.3. Insomnia Symptoms

Symptoms of insomnia were assessed using the insomnia severity index (ISI), which has adequate internal consistency (Cronbach’s α = 0.74) [24,25]. The ISI is a brief self-administered scale that evaluates personal perception of insomnia on a 4-points Likert scale (range 0–28; cut-off points of 8, 15 and 22 that indicate the presence of subthreshold, moderate, and severe insomnia).

#### 2.2.4. Posttraumatic Stress Disorder symptoms

To assess the perceived emotional distress that accompanies a stressful life event, we used the impact of event scale–revised (IES–R), that showed a high internal consistency (Cronbach’s α = 0.95) [26,27,28]. Our participants were forced to choose the pandemic as the stressful event investigated. The scale is a self-administered instrument made up of twenty-two items divided into three scales (intrusion, avoidance and hyperarousal), evaluated by participants using a five-point Likert scale (range 0–88). In relation to PTSD, a score of 33 was considered as the cut-off point.

#### 2.2.5. Burnout Symptoms

Symptoms of burnout were assessed with the Maslach burnout inventory (MBI) and cut-off criteria for Italian HW were used [29,30]. It is a self-administered scale formed by twenty-two items with seven response options on a Likert scale from zero (never) to six (every day), which creates three different components of the burnout: emotional exhaustion (EE, total range 0–54), depersonalization (DP, total range 0–30) and personal accomplishment (PA, total range 0–48). MBI score ranges were 0–14, 15–23, and 24–54 for the emotional subscale and indicate low, moderate, and high levels of EE; for depersonalization, the subscale score ranges were 0–3, 4–8 and 9–30, indicating low, moderate, and high levels of DP; finally, for the reduced personal accomplishment subscale, score ranges were 48–37, 36–30, and 29–0, indicating the presence of low, moderate and high levels of PA. MBI has shown good enough reliability for EE scale (Cronbach’s α = 0.88) and a lower reliability for DP (Cronbach’s α = 0.71) and PA scales (Cronbach’s α = 0.76) [31].

### 2.3. Data Analysis

We reported the percentages of participants who rated low to high scores in psychological distress. We used a multivariate multiple regression analysis to examine the effect of demographic characteristics, personal exposure to COVID-19, and burnout on levels of depression, anxiety, insomnia, and PTSD, in order to underline the risk factors for developing stress response symptomatology and other forms of psychopathology. Then we divided the sample into two groups with low and medium/high levels of burnout and we used the two-sample T test to investigate the presence of statistically significant differences between those two groups in presenting symptoms of depression, anxiety, insomnia, or PTSD.

All statistical analyses were performed using the SPSS version 25.0 (IBM Corp., Armonk, NY, USA), and the value of statistical significance was set at *p* < 0.05 (two tailed). We used a listwise deletion for the missing data.

## 3. Results

### 3.1. Sample Characteristics

A total number of 731 participants completed the screening (29.7% of the HW employed in the hospitals enrolled in our study). Sociodemographic characteristics of the sample and mental health measures are reported in Table 1. The mean age of our population was 48.45 ± 9.81. The majority of the sample (76%; *n* = 556) were female and 671 respondents (92%) reported to be directly exposed to covid-19. More than half of the population (62%; *n* = 451) thought that work impacted on their psychological wellbeing and the need for psychological support was perceived by 40% of the sample (*n* = 296). A total number of 297 respondents declared experiencing sleeping (21%) and anxiolytic medications intake (20%) and 32% (*n* = 234) declared a customary alcohol consumption. More than half of the sample (62%; *n* = 453) presented mild (32%) to severe (13%) depressive symptoms, with a PHQ total mean score of 7.34, whereas 447 participants (61%) reported having anxiety symptoms, being severe in 11% of the sample, with a GAD-7 mean score of 6.92. Of the 731 participants, 349 (48%) experienced symptoms of insomnia, with an ISI mean total score of 8.25. Lastly, more than a third of our sample (34%; *n* = 250) was experiencing post-traumatic symptomatology and nearly half of the sample was facing increased levels of burnout, especially EE was perceived by 37% of the participants (*n* = 270).

### 3.2. Identification of Post-Traumatic Symptoms and Other Psychopathology Risk Factors

We searched if some of the considered variables had a direct influence on PTSD (as measured by IES–R), depression (as measured by PHQ9), anxiety (as measured by GAD 7) or insomnia (as measured by ISI) with multivariate multiple regression analyses (data are reported in Table 2).

We found that male gender was a protective factor in developing PTSD, having a negative association with post-traumatic symptomatology (Std. β = −0.28, *p* = 0.006); similarly, seniority over 30 years had a negative association with depressive symptoms (Std. β = −0.02, *p* = 0.046). On the other hand, the need for psychological support was associated with higher levels of post-traumatic symptomatology (Std. β = 0.56, *p* < 0.001), depressive symptoms (Std. β = 0.16, *p* < 0.001), anxiety (Std. β = 0.13, *p* < 0.001), and insomnia (Std. β = 0.18, *p* < 0.001); while the perception of work impacting on wellbeing was associated with post-traumatic symptomatology (Std. β = 0.52, *p* = 0.001), and anxiety (Std. β = 0.06, *p* = 0.043).

We also found a positive association between the use of sleeping medication and depression (Std. β = 0.07, *p* = 0.024) and insomnia (Std. β = 0.20, *p* < 0.001); while the use of anxiolytic drugs was linked to post-traumatic (Std. β = 0.24, *p* = 0.026), depressive (Std. β = 0.11, *p* < 0.001), and anxiety symptoms (Std. β = 0.09, *p* = 0.002).

Lastly, looking at the correlation between burnout and psychopathology, we found that EE was associated with PTSD (Std. β = 1.18, *p* < 0.001), depression (Std. β = 0.47, *p* < 0.001), anxiety (Std. β = 0.48, *p* < 0.001), and insomnia (Std. β = 0.47, *p* < 0.001); while low scores on the PA subscale were associated with higher depressive (Std. β = −0.10, *p* < 0.001) and anxiety symptoms (Std. β = 0.02, *p* = 0.005).

### 3.3. Burnout Impact on Health Workers Mental Health

In Table 3, we report descriptive statistics for the instruments used to evaluate the presence of psychopathology. We divided the sample into low and moderate–high risk of burnout reporting the mean score for each scale of psychopathology (PHQ-9, GAD7, ISI and IES-R) stratified for each burnout subscale. The last column of the table reports results of the two-sample t-test showing the risk of developing clinical symptomatology based on the levels of burnout. Results demonstrate that medium/high scores in all burnout subscales have a statistically significant association with insomnia, depression, anxiety, and post-traumatic symptoms (*p* < 0.001). EE appears to have the strongest impact on the whole range of the psychopathological presentation (Cohen’s d > 0.80), being a strong predictor of future symptomatology; whereas the other subscales (DP and PA) show only a moderate impact (Cohen’s d = 0.50–0.79), with the only exception of PA having a low impact on insomnia symptomatology (Cohen’s d = 0.48).

## 4. Discussion

The aim of our survey was to investigate the mental health wellbeing of Italian HW during the first outbreak of the COVID-19 pandemic. We analyzed the presence of depression, anxiety, insomnia, and post-traumatic symptomatology, as the literature describes in these emergency situations [32]; and we tried to find major and specific risk factors implied in the development of psychopathology.

We have also looked to see if burnout, already very common among HW, had a specific role in promoting or worsening the symptomatology.

As other studies show, we found high levels of mental distress, especially elevated levels of anxiety and depression in more than half of our participants (62% and 61% respectively) [33]. Our results show some slight differences from international studies, with a higher rate of depression compared with other research and, when compared to a recent review reporting PTSD prevalence among HW during COVID-19 pandemic, lower post-traumatic symptomatology [34,35]. However, other findings coincide with our observations, such as a study from Bassi and colleagues that found a prevalence of PTSD of 39.8% on HW in Lombardy [36].

We also observed that 62% of participants believed that work had an important impact on their psychological wellbeing and even if we did not find a strong correlation between COVID-19 specific factors and psychopathology, we reported that, in our sample, the feeling of work impacting on one’s own psychological wellbeing was associated with post-traumatic symptomatology (*p* < 0.001, Table 2); as we can see in other studies exploring the relationship between the pandemic and work impact [37].

Almost 40% of HW felt the need for psychological support; previous literature highlighted how the perception of needing psychological support was strongly associated with higher levels of psychological distress and also in our study it was a strong predictor for all the psychopathology examined (Table 2) [38].

As well as in other studies, male gender and work seniority turned out to be protective factors against the development of depression, but not other mental health disorders [39].

Regarding burnout, in our sample we found a moderate/severe presence of burnout, with high scores in each subscale, above all in EE (Table 1). Our findings agree with other observations [40,41]; in particular a recent review from Sanghera and colleagues found a prevalence of burnout between 3.1% to 43.0% among HW during COVID-19 and Barello and colleagues found high levels of EE in 37% of the participants, from an Italian sample of HW directly employed in COVID-19 units [42,43].

To our knowledge, this is the first study investigating the correlation between levels of burnout and the risk for other mental health disorders during the COVID-19 pandemic. We found burnout was an important predictor of psychological sufferance during COVID-19 outbreak among healthcare professionals. Particularly, we found a linear association between levels of EE and all the psychopathology investigated (depression, anxiety, insomnia, and post-traumatic symptoms), whereas PA was associated with higher levels of anxiety and depression. Moreover, when our sample was divided by the presence of moderate/severe burnout, we could underline a strong association between all the subscales of burnout and other psychopathology, with EE was confirmed as the most important predictor of psychological sufferance among HW.

Our findings fit with the theoretical model of burnout, where among the three dimensions of burnout, EE appears to be the most predictive factor for stress-related symptomatology, both somatic (headaches, somatic fatigue, gastrointestinal disorders) and psychological [44]. In fact, recent and previous studies on HW health during pandemic outbreaks (COVID-19, severe acute respiratory syndrome (SARS), Middle East respiratory syndrome (MERS), and H1N1 influenza) demonstrate that burnout is associated with increased risk of manifesting somatic and psychic symptoms [43,45] and it has a direct impact on stress-related symptomatology, anxiety, mood disorders, substance abuse, suicides and also on professional performance, early retirements or unexpected resignations [46].

Between all of burnout’s dimension, EE is the first to develop and this could explain its higher prevalence, whereas detachment and cynicism, correlated to other subscales, often appear later [45].

These results require special attention, as previous studies showed that EE is frequently associated with suboptimal patients’ care and professional inefficiencies along with a long-lasting effect on health professionals’ health status [47,48].

From the perspective of clinical implication, we aimed to detect psychological distress among HW in order to offer stepped care support to prevent the development of severe psychopathology and to maintain an optimal level of patients’ care during the pandemic. Even outside the COVID-19 emergency, taking care of HW’s levels of burnout could lead to better job performances. For these reasons, at the end of the survey we gave information about mental health support and care that were given within the hospitals enrolled in the study, in order to decrease their employees’ levels of sufferance.

Our survey was conducted almost twelve-to-sixteen weeks after the first peak of the pandemic in Italy; this could be seen as a bias, taking into account that other factors not specifically inquired (such as safety precautions, the presence of protective equipment that lacked during the first weeks of the pandemic, the quarantine, etc.) could have affected the presentation of symptomatology, and the delay in the data collection could have missed the acute stress of the first phase of the pandemic. Nevertheless, our results are consistent with studies that highlight that during the initial phase of the outbreak, the symptoms of anxiety and acute stress usually prevailed, whether during the “repair” phase, when the infection starts to be under control, usually symptoms of depression and avoidance arise [49].

Another limit to our study is that, even if the survey was submitted to the all the HW employed in COVID-19 hospitals and the majority of them (68%) had worked with COVID-19 patients, we assumed that a higher percentage of acute stress and post-traumatic stress symptomatology could be found more likely in professionals working in specific wards (as intensive and sub-intensive care units or emergency departments). Regardless, we decided not to investigate the specific workplace in order to increase the perception of privacy and confidentiality involved in the survey compilation.

Moreover, the use of self-report instruments is a limit to our research, which allowed us to ensure privacy and to reach a bigger number of HW through the platform, but we were not able to compare the data with previous information on participants’ health records to see if there were professionals with a higher risk to develop mental health illnesses.

Furthermore, we could not collect the baseline levels of burnout, even if we run previous surveys regarding levels of burnout in HW, but only in specific professional categories. We need to compare the basal levels of burnout with those that arise in emergency situations, in order to follow changes and the development of the severity of the presentation and their association with symptomatology over time. Additionally, further longitudinal research should enquire about the role of burnout in developing psychiatric symptomatology among HW.

Lastly, these findings need to be replicated in larger samples, in fact only a third of HW working in the hospitals enrolled in our study responded to our survey, and it should be compared to symptomatology in the general population.

All the associations studied are in the expected direction: people who feel their job have an impact on their wellbeing and they are more willing to seek psychological support, show higher levels of burnout with a more cynical and detached attitude towards patients and colleagues, and are likely to present depression, anxiety, and post-traumatic symptoms, and insomnia. These findings support the usefulness and suitability of our project, integrated with our survey study, to offer stable, worker-friendly, fitting with the work schedule, psychological and psychiatric support in order to decrease the burden and the sufferance that this pandemic has inflicted on HW [50,51,52,53,54]. It is important to embed in the workplace targeted and specific prevention strategies, especially in healthcare settings, in order to decrease levels of burnout, to improve mental health wellbeing and resilience, and to ensure a safe and supportive environment that leads to better psychological and physical wellbeing.

## 5. Conclusions

The rapid spread of the COVID-19 pandemic had shown how HW could present high levels of psychological distress and burnout, and the latter was found to be an important predictor of sufferance.

This pandemic has enlightened the need for stable psychological support for HW, not only during the peak of the pandemic, when the work overload and other factors that impact on HW mental health are more defined, but also in everyday situations that already challenge HW regarding their mental and physical wellbeing, as literature on burn-out indicates.

## Figures and Tables

**Table 1:** Study sample characteristics (*n* = 731).

Main Categories	Variable		Frequencies	Percent
Socio- Demographic	Gender	Female	556	76
		Male	175	24
	Marital status	Married/Common law	483	66
		Stable relationship (not living together)	69	10
		Separated/divorced	89	12
		Single	90	12
	Living alone	No	596	18
		Yes	135	82
	Offspring	No	253	65
		Yes	478	35
Employment	Job	Medical staff	192	26
		Nurse	359	49
		Social health worker	72	10
		Other	108	15
	Seniority	Less than 10 years	197	27
		Between 11 and 20 years	172	24
		Between 21 and 30 years	175	24
		Over 30 years	187	25
COVID-19 specific stressors	Working with COVID-19 positive patients	No	232	32
		Yes	499	68
	Have you changed your ward?	No	577	79
		Yes	154	21
	Have you been exposed to covid-19?	No	60	8
		Yes	671	92
	Have you been infected?	No	549	75
		Yes	114	16
		Other	68	9
	Beloved with COVID-19	No	386	53
		Yes	345	47
	Beloved dead with COVID-19	No	587	80
		Yes	144	20
Other psychological variables	Work impact on psychological well being	No	280	38
		Yes	451	62
	Need for psychological support	No	435	60
		Yes	296	40
	Use of sleeping medication	No	579	79
		Yes	152	21
	Use of anxiolytic medication	No	586	80
		Yes	145	20
	Use of alcohol	No	497	68
		yes	234	32
Psychological Screening	PHQ9	None	278	38
		Mild	233	32
		Moderate	126	17
		Moderately severe	71	10
		Severe	23	3
	GAD7	None	284	39
		Mild	246	34
		Moderate	122	16
		Severe	79	11
	ISI	None	382	52
		Minimal or none	220	30
		Moderate	105	14
		Severe	24	4
	IESR Tot	None	481	66
		Possible PTSD	250	34
	MBI—Exhaustion	Low	334	46
		Medium	127	17
		High	270	37
	MBI—Depersonalization	Low	379	52
		Medium	192	26
		High	160	22
	MBI—Personal Accomplishment	Low	384	52
		Medium	202	28
		High	145	20

PHQ-9 = Patient health questionnaire-9; GAD-7 = generalized anxiety disorder-7; ISI = insomnia severity index; IES-R = impact of event scale–revisedMBI = Maslach burnout inventory.

**Table 2:** Results of Multivariate Multiple Regression.

Psychopatology	IES–R Tot			PHQ9			GAD7			ISI		
Predictor	β(SE)	t	*p*-Value	β(SE)	t	*p*-Value	β(SE)	t	*p*-Value	β(SE)	t	*p*-Value
(Intercept)	13.98 (5.18)	2.70	0.001 **	4.48 (1.45)	3.08	0.002 *	5.05 (1.38)	3.67	<0.001 ***	2.77 (1.78)	1.56	0.119
Age	−0.04 (0.09)	−0.52	0.600	0.03 (0.02)	1.32	0.188	−0.02 (0.02)	−1.03	0.305	0.03 (0.03)	1.17	0.244
Gender—male	−3.56 (1.29)	−2.75	0.006 **	−0.64 (0.36)	−1.76	0.079	−0.65 (0.34)	−1.89	0.058	−0.10 (0.44)	−0.23	0.819
Job—Medical staff	0.59 (1.69)	0.34	0.731	−0.02 (0.48)	−0.05	0.958	0.38 (0.45)	0.85	0.397	−0.43 (0.58)	−0.74	0.459
Job–Nurse	−0.32 (1.55)	−0.21	0.838	−0.63 (0.44)	−1.45	0.149	−0.27 (0.41)	−0.67	0.506	−0.32 (0.53)	−0.60	0.548
Job—Social Health worker	0.71 (2.19)	0.32	0.747	−0.48 (0.62)	−0.79	0.431	0.57 (0.58)	0.98	0.329	−1.07 (0.75)	−1.42	0.156
Seniority—11–20 years	−1.28 (2.22)	−0.58	0.565	−0.87 (0.62)	−1.40	0.162	−0.01 (0.59)	−0.02	0.981	−0.27 (0.76)	−0.36	0.718
Seniority—21–30 years	−1.89 (1.69)	−1.12	0.263	−0.62 (0.47)	−1.31	0.191	0.04 (0.49)	0.09	0.924	−0.52 (0.58)	−0.89	0.369
Seniority—Over 30 years	0.93 (1.94)	0.48	0.633	−1.09 (0.55)	−1.99	0.046 *	−0.36 (0.51)	−0.69	0.489	−0.39 (0.66)	−0.59	0.549
COVID-19 specific stressors	0.57 (1.94)	0.29	0.770	−0.57 (0.54)	−1.06	0.289	0.19 (0.51)	0.39	0.699	1.23 (0.66)	1.86	0.064
Infected No	0.65 (1.93)	0.34	0.736	−0.45 (0.54)	−0.83	0.406	−0.70 (0.51)	−1.38	0.169	0.29 (0.66)	0.44	0.663
Infected Yes	1.33 (2.25)	0.59	0.554	0.24 (0.64)	0.38	0.705	−0.41 (0.59)	−0.69	0.488	0.38 (0.77)	0.50	0.617
Beloved with covid—Yes	0.53 (1.25)	0.42	0.674	0.19 (0.35)	0.56	0.579	0.16 (0.33)	0.48	0.633	−0.11 (0.43)	−0.25	0.803
Beloved dead with covid—Yes	1.59 (1.56)	1.02	0.307	−0.35 (0.44)	−0.79	0.431	0.21 (0.41)	0.52	0.604	0.22 (0.53)	0.41	0.681
Work impact on wellbeing—Yes	5.73 (1.29)	4.45	<0.001 ***	0.45 (0.36)	1.26	0.209	0.69 (0.34)	2.03	0.043 *	0.46 (0.44)	1.05	0.292
Need for psychological support—Yes	7.19 (1.29)	5.58	<0.001 ***	1.91 (0.36)	5.28	<0.001 ***	1.71 (0.34)	4.99	<0.001 ***	2.11 (0.44)	4.77	<0.001 ***
Sleeping medication—Yes	2.72 (1.49)	1.84	0.067	0.94 (0.42)	2.27	0.024 *	0.61 (0.39)	1.54	0.124	2.71 (0.51)	5.33	<0.001 ***
Anxiolytic medication—Yes	3.43 (1.54)	2.23	0.026 *	1.86 (0.43)	4.31	<0.001 ***	1.31 (0.41)	3.22	0.002 **	0.77 (0.53)	1.46	0.145
Alcohol—Yes	−0.46 (1.19)	−0.39	0.698	−0.02 (0.33)	−0.05	0.963	0.09 (0.32)	0.31	0.759	−0.01 (0.41)	0.00	0.999
MBI Depersonalization	0.18 (0.11)	1.66	0.098	−0.01 (0.03)	−0.37	0.711	−0.01 (0.02)	−0.07	0.947	−0.07 (0.04)	−1.82	0.069
MBI Personal Accomplishment	−0.09 (0.06)	−1.37	0.172	−0.06 (0.01)	−3.50	<0.001 ***	−0.05 (0.02)	−2.79	0.005 **	−0.07 (0.02)	−3.01	0.002 **
MBI—Emotional Fatigue	0.45 (0.05)	8.94	<0.001 ***	0.19 (0.01)	13.91	<0.001 ***	0.18 (0.01)	13.63	<0.001 ***	0.19 (0.02)	11.31	<0.001 ***
R 2 (adjusted R 2)	0.49 (0.47)			0.57 (0.55)			0.56 (0.55)			0.46 (0.44)		
Residual std. error	13.56			3.81			3.60			4.65		
F statistic (df = 21, 672)	30.21 ***			41.62 ***			40.74 ***			27.38 ***		

IES–R = Impact of event scale–revised; PHQ-9 = patient health questionnaire-9; GAD-7 = generalized anxiety disorder-7; ISI = insomnia severity index; SE = standard error; *** = significant for *p* < 0.001, ** = significant for *p* < 0.01., * = significant for *p* < 0.05.

**Table 3:** Descriptive statistics for measured variables and t-tests for burnout differences.

Variable	Mean and Standard Deviation	MBI	Mean and Standard Deviation (Low Risk)	Mean and Standard Deviation (Medium and High Risk)	*t*-Test for Risk of Burnout Differences
PHQ9	7.32 ± 5.69	DP	5.70 ± 4.90	9.10 ± 5.96	t(638.19) = −8.17, *p* < 0.001 *** Cohen’s d = 0.62
		EE	3.78 ± 3.48	10.28 ± 5.48	t(647.77) = −18.9, *p* < 0.001 *** Cohen’s d = 1.39
		PA	5.61 ± 4.66	9.23 ± 6.11	t(606.46) = −8.67, *p* < 0.001 *** Cohen’s d = 0.67
GAD7	6.86 ± 5.84	DP	5.35 ± 4.74	8.53 ± 5.49	t(653.49) = −8.14, *p* < 0.001 *** Cohen’s d = 0.62
		EE	3.53 ± 3.23	9.65 ± 5.17	t(643.54) = −18.98, *p* < 0.001 *** Cohen’s d = 1.39
		PA	5.43 ± 4.74	8.47 ± 5.53	t(646.39) = −7.72, *p* < 0.001 *** Cohen’s d = 0.59
ISI	8.19 ± 6.23	DP	6.80 ± 5.58	9.73 ± 6.55	t(649.6) = −6.31, *p* < 0.001 *** Cohen’s d = 0.48
		EE	4.63 ± 4.22	11.14 ± 6.08	t(672.78) = −16.44, *p* < 0.001 *** Cohen’s d = 1.22
		PA	6.59 ± 5.71	9.98 ± 6.32	t(661.09) = −7.39, *p* < 0.001 *** Cohen’s d = 0.56
IES–R	26.6 ± 18.62	DP	20.93 ± 15.23	32.85 ± 19.99	t(612.54) = −8.77, *p* < 0.001 *** Cohen’s d = 0.68
		EE	16.38 ± 13.06	35.14 ± 18.28	t(676.17) = −15.72, *p* < 0.001 *** Cohen’s d = 1.16
		PA	22.05 ± 16.75	31.7 ± 19.31	t(649.62) = −6.99, *p* < 0.001 *** Cohen’s d = 0.54

MBI = Maslach burnout inventory; DP = depersonalization; EE = emotional exhaustion; PA = personal accomplishment; PHQ-9 = patient health questionnaire-9; GAD-7 = generalized anxiety disorder-7; ISI = insomnia severity index; IES–R = event scale–revised; *** = significant for *p* < 0.001.

## Data Availability

The data presented in this study are available on request from the corresponding author. The data are not publicly available due to HW’ privacy.
